# Clinical Outcomes of Surgical Treatments for Primary Malignant Bone Tumors Arising in the Acetabulum

**DOI:** 10.1155/2015/430576

**Published:** 2015-09-16

**Authors:** Tomohiro Fujiwara, Koichi Ogura, Eisuke Kobayashi, Yoshikazu Tanzawa, Fumihiko Nakatani, Hirokazu Chuman, Akira Kawai

**Affiliations:** Division of Musculoskeletal Oncology, National Cancer Center Hospital, 5-1-1 Tsukiji, Chuo-ku, Tokyo 104-0045, Japan

## Abstract

The functional and oncologic results of eighteen patients with primary malignant periacetabular tumors were reviewed to determine the impact of surgical treatment. The reconstruction procedures were endoprosthesis (11), hip transposition (4), iliofemoral arthrodesis (2), and frozen bone autograft (1). After a mean follow-up of 62 months, 13 patients were alive and 5 had died of their disease; the 5-year overall survival rate was 67.2%. The corresponding mean MSTS scores of patients with endoprosthesis (11) and other reconstructions (7) were 42% and 55% (49%, 68%, and 50%), respectively. Overall, postoperative complications including deep infection or dislocation markedly worsened the functional outcome. Iliofemoral arthrodesis provided better function than the other procedures, whereas endoprosthetic reconstruction demonstrated poor functional outcome except for patients who were reconstructed with the adequate soft tissue coverage. Avoiding postoperative complications is highly important for achieving better function, suggesting that surgical procedures with adequate soft tissue coverage or without the massive use of nonbiological materials are preferable. Appropriate selection of the reconstructive procedures for individual patients, considering the amount of remaining bone and soft tissues, would lead to better clinical outcomes.

## 1. Introduction

**Figure 1:**
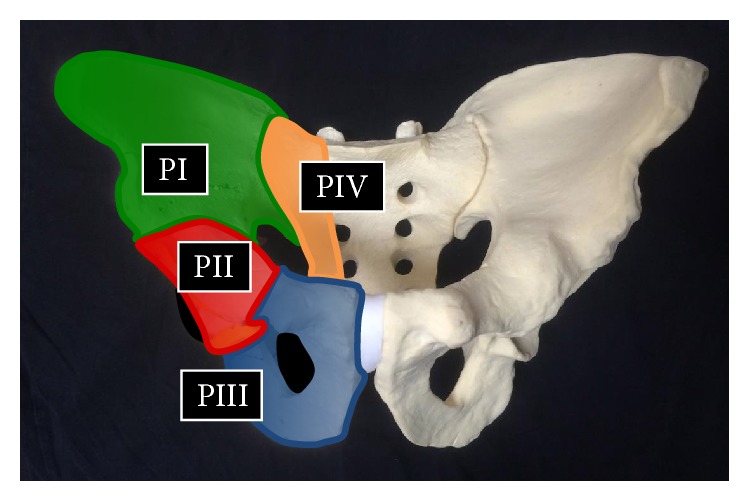
Diagram showing the resected area according to the classification system of Enneking and Dunham.

Although primary sarcomas arising in the pelvis are relatively rare, surgical treatment for these diseases remains difficult. Resections of pelvic bone are classified according to the system of Enneking and Dunham (Figure 1) [1, 2]. PII or periacetabular resections present a unique surgical challenge in that no specific form of reconstruction has proved superior [3]. There is still much debate about the best reconstructive option for these patients, and many such options exist. These include endoprosthetic reconstruction [4–6], hip transposition [7, 8], iliofemoral arthrodesis [9, 10], biologic reconstruction (using allografts or autografts from the tibia, fibula, iliac crest, or pelvis) [11, 12], or hip rotationplasty [13]. However, there is still no standard procedure for reconstruction after resection of malignant periacetabular tumors. Indeed, functional outcomes in patients who have undergone periacetabular resections also vary greatly depending on the type of reconstruction [14]. In several published series, musculoskeletal tumor society (MSTS) scores have been within the range of 40–60% [9, 15]. The major problem associated with these reconstructive procedures is their high rate of postoperative complications, such as delayed wound healing, infection, dislocation, aseptic loosening, or local recurrence [14–16]. Published series have reported complication rates of 18–65% depending on the reconstructive procedure employed [6]. The appropriateness of any reconstructive procedure needs to be decided on the basis of its functional results, as well as its rate of associated complications.

Over the past 10 years, a variety of reconstructive procedures have been employed at our institution. The purpose of the present study was to evaluate the surgical outcome, including complication rate and functional score, in patients after acetabular resection according to the surgical procedures employed, and to determine the clinical and functional outcome after resection of periacetabular tumors.

## 2. Patients and Methods

We retrospectively reviewed 18 patients with primary periacetabular bone tumors who underwent acetabular resection and reconstruction between June 1996 and December 2012. Their clinical data, treatment modalities, and treatment outcome were reviewed retrospectively by reference to the medical records. The following data were examined: demographic data (patient age at operation, gender, tumor size, and histologic diagnosis), surgical details (reconstructive procedures, lesion resected, and surgical margins), lesion resected, adjuvant therapy (chemotherapy and radiotherapy), postoperative complications (e.g., infection or dislocation), oncologic outcomes, and functional outcomes.

Acetabular lesions were resected using a variety of procedures according to the classification system of Enneking and Dunham (Figure 1). Surgical margins were evaluated on the basis of surgical and pathological reports according to the system described by Enneking [17]. Limb-sparing surgery was performed when the surgical margin would be comparable to that of external hemipelvectomy, as well as when adequate soft tissue and the sciatic nerve could be preserved during tumor resection. The reconstruction procedures after periacetabular tumor resection included endoprosthetic reconstruction (11), hip transposition (4), iliofemoral arthrodesis (2), and reconstruction using frozen bone autograft (1). The decision on the reconstruction procedure was made according to patient opinion based on the detailed informed consent obtained by the staff surgeon, including the characteristics of each procedure that had been reported [14, 15, 18–21]. For patients who underwent endoprosthetic reconstruction, we used an endoprosthetic system with a constrained hip mechanism (C-THA; Hip Reconstruction Cup; Japan Medical Materials) to obtain iliofemoral stability [6, 22], which was composed of a cup with a plate to anchor prosthesis into the remaining ilium. Hip transposition, iliofemoral arthrodesis, and reconstruction using frozen bone autograft were performed as previously reported [6–8, 10, 22–24].

Function was assessed at the final follow-up using the MSTS system developed by Enneking et al. [25]. The MSTS system is based on the analysis of factors pertinent to the patient as a whole (pain, restriction of activities and/or occupation, and emotional acceptance) and factors specific to the affected limb (the use of walking supports, walking distance, and gait). Each parameter is given a value ranging from 0 to 5, according to specific criteria, and the overall result is expressed as a summation of all the parameters, which then is converted to a percentage of the maximum possible score. We defined postoperative complications requiring surgical interventions within one year as major complications and all other complications as minor. Patient survivals were estimated by the Kaplan-Meier method calculated from the date of definitive surgery to the time of patient death or the last follow-up for survivors.

Ethical approval was obtained from the Institutional Review Boards of National Cancer Center Hospital.

## 3. Results

### 3.1. Oncological Results for the Entire Group

Patient demographics and treatment data are summarized in Table 1. There were 13 males and 5 females with a mean age of 41 years (range, 8–69 years) at the time of surgical treatment. The mean tumor size was 11.7 cm (range, 7–20 cm). According to histological distribution, the primary tumor was recorded as osteosarcoma in 8 patients, chondrosarcoma in 5, malignant fibrous histiocytoma (MFH) of bone in 2, Ewing sarcoma in 2, and fibrosarcoma of bone in 1. The mean follow-up period was 62 months (range, 8–155 months). In this series, 11 patients received neoadjuvant chemotherapy and no patient received radiation therapy preoperatively. After surgery, 9 patients received adjuvant chemotherapy and 1 patient received radiation therapy.

The acetabular lesions were resected using a variety of procedures according to the classification system of Enneking and Dunham [1, 2]: type PII resection in 2 cases, type PI-II resection in 4 cases, type PII-III resection in 8 cases, and type PI-II-III resection in 4 cases (Table 1).

The surgical margins in this study group were classified as wide in 17 patients and intralesional in 1 patient (Table 1). One patient whose surgical margin was intralesional was reconstructed with endoprosthesis. This patient with dedifferentiated chondrosarcoma suffered local recurrence at 12 months after the operation, developed lung metastasis at 13 months, and died of the disease at 16 months after surgery.

**Figure 2:**
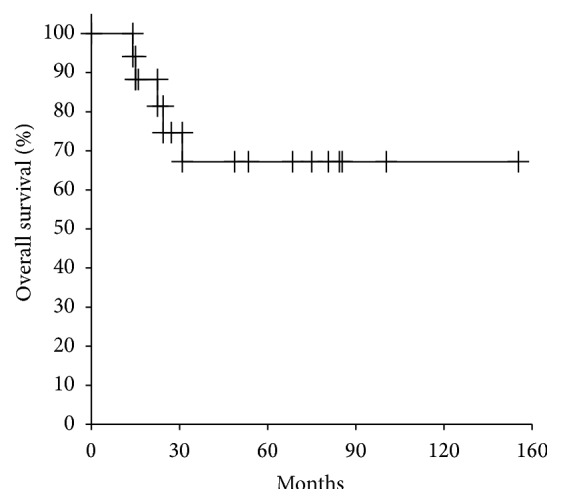
Cumulative overall survival curve for all patients estimated by the Kaplan-Meier method.

After a mean overall follow-up of 62 months (range, 8–155 months), 11 patients (61%) had no evidence of disease (NED), 2 (11%) were alive with disease (AWD), and 5 (28%) had died of the disease (DOD) (Table 1). The 5-year overall survival rates were 67.2% (Figure 2).

### 3.2. Complications according to Surgical Procedures

Postoperative complications are listed in Table 2.

After endoprosthesis replacement, most patients suffered postoperative complications. Among 11 patients who underwent endoprosthesis reconstruction, 6 (55%) had major complications, which required surgical interventions. The complications comprised superficial infection in 2 patients (18%), deep infection in 2 (18%), wound complication in 3 (27%), dislocation in 2 (18%), abdominal hernia in 1, and local recurrence in 1. Eight additional surgical procedures were performed in patients of this group, including 6 revisions for deep infection or wound complication and 2 for implant dislocation. No complications developed in 5 patients whose gluteus medius or gluteus maximus was preserved, or in those who underwent coverage of the large soft tissue defect with a rectus abdominis myocutaneous flap [26].

On the other hand, among those who performed other reconstructions than endoprosthesis, only one patient (14%) experienced major complication, which required surgical intervention. After hip transposition in 4 patients, one suffered postoperative infection, and all of the patients had leg-length discrepancy. One patient required additional surgical procedure for deep infection and wound problem. This patient underwent reconstruction with a Gore-Tex sheet around the iliac resection site, which was considered to have caused postoperative infection.

Of the 2 patients who underwent iliofemoral arthrodesis, both had leg-length discrepancy and one suffered implant complication. One patient suffered screw breakage at 2 years after surgery, which required an additional fixation. Later the patient underwent a limb-lengthening operation for leg-length discrepancy at another institution. The other patient died of metastatic disease at 2 years after surgery.

One patient who underwent reconstruction with a frozen bone autograft showed delayed postoperative wound healing. At 1 year after surgery, the patient presented with hip osteoarthritis, which has since shown gradual progression.

### 3.3. Functional Results according to the Surgical Procedures

The mean functional score in the present series, according to the MSTS system, was 14.2 points (47%) at the latest follow-up. The functional results according to the various surgical procedures employed are shown in Table 3.

**Figure 3:**
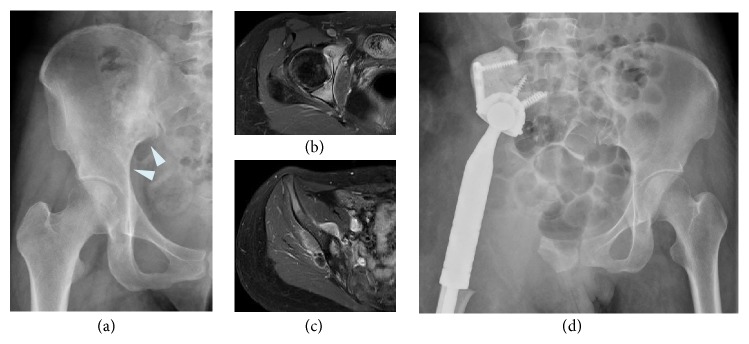
(a) Preoperative anteroposterior radiograph of the pelvis of a 28-year-old female who had osteosarcoma of the acetabulum and ilium. ((b) and (c)) Gadolinium-enhanced axial T1-weighted MRI showing the tumor arising in the acetabulum and ilium. After neoadjuvant chemotherapy, the patient underwent tumor wide resection and endoprosthetic reconstruction with no postoperative major complications. (d) Postoperative radiograph 3 years after endoprosthetic reconstruction.

The 11 patients who underwent endoprosthetic reconstruction had a mean functional score of 12.5 points (42%) out of a maximum of 30 points (range, 13–70%). All patients were unable to walk without walking aids; no patient had a score of >3 points (out of a possible 5 points). Eight patients required constant pain medication with nonnarcotic analgesics (four with moderate pain and four with mild pain). The mean emotional acceptance score was 46% (range, 0–60%). Notably, the mean MSTS score for the six patients without postoperative deep infection or dislocation was 55% (Figure 3), whereas that for the other five patients who suffered these complications was 28%.

**Figure 4:**
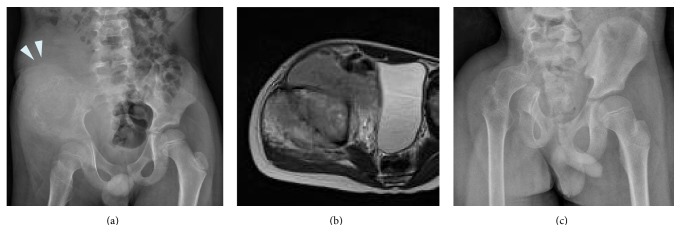
(a) Preoperative anteroposterior radiograph of the pelvis of an 8-year-old boy who had Ewing sarcoma in the ilium and acetabulum. (b) Axial T2-weighted MR image showing the tumor arising in the acetabulum. (c) Postoperative radiograph one year after hip transposition.

The 4 patients who underwent reconstruction with hip transposition had a mean functional score of 49% (range, 33–67%). They complained of the least amount of pain. Only one patient required continuous use of analgesic medications. Notably, the mean emotional acceptance score was 70% (range, 40–100%), which was the highest among all the groups. All of these patients had limb-length discrepancy (Figure 4) and required a shoe lift. However, their walking ability was comparable to that of patients who underwent other surgical procedures.

**Figure 5:**
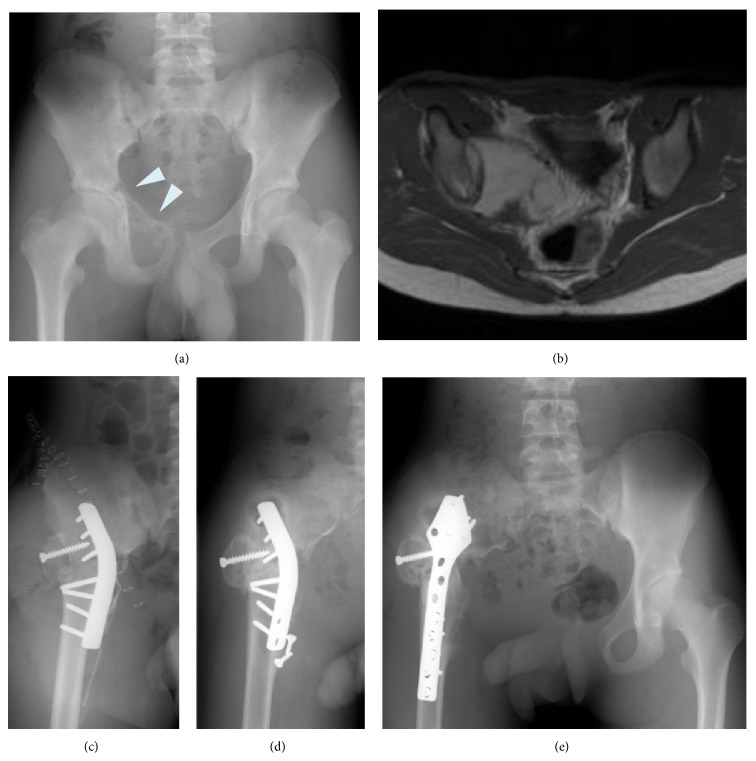
(a) Preoperative anteroposterior radiograph of the pelvis of a 14-year-old boy who had Ewing sarcoma of the acetabulum and pubis. (b) Axial T2-weighted MRI showing the tumor arising in the acetabulum. (c) Postoperative radiograph showing plate fixation of the proximal femur to the remaining ilium after PII-III resection. (d) Plain radiograph showing screw breakage 2 years after first iliofemoral arthrodesis. (e) Plain radiograph 11 years after refixation.

Two patients who underwent iliofemoral arthrodesis had a mean functional score of 68%. One patient had no pain, and the other had mild pain. The mean emotional acceptance score was 50%. One patient required no support for walking (Figure 5), while the other was able to walk with one crutch. Both patients had limb-length discrepancy and required a shoe lift. One of them underwent additional limb-lengthening surgery after long-term survival and has maintained a high MSTS score.

**Figure 6:**
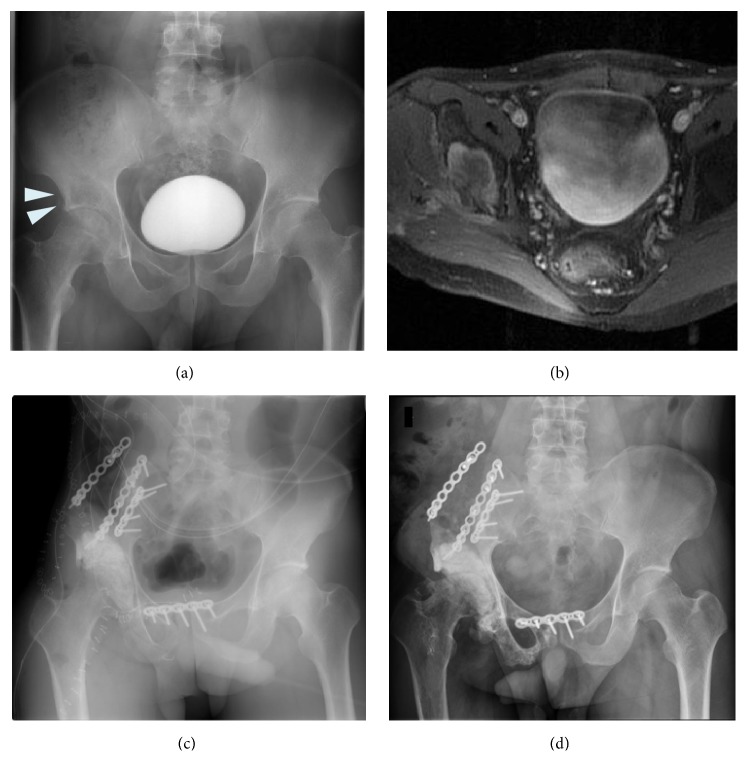
(a) Preoperative anteroposterior radiograph of the pelvis of a 38-year-old man who had MFH of bone in the acetabulum. (b) Gadolinium-enhanced axial T1-weighted MRI showing the tumor arising in the acetabulum. (c) Postoperative radiograph after reconstruction using a frozen bone autograft. (d) Follow-up radiograph 1.5 years after surgery showing osteoarthritis of the hip joint.

The patient who was reconstructed with a frozen bone autograft had a functional score of 50%. He complained of moderate pain, which seemed to be caused by osteoarthritis, and used nonnarcotic analgesics (Figure 6). Two crutches were necessary for walking, and his emotional acceptance score was 20%.

Consequently, the mean functional score for patients with endoprosthesis (42%) was worse than that for patients with other reconstructions (55%), which seemed to be attributed to the high complication rate in the former group. The best results were obtained in patients who underwent iliofemoral arthrodesis, although the number of patients was small. Regardless of the surgical procedures, the functional scores for patients with postoperative deep infections or dislocations were quite low; the mean MSTS scores for patients with these complications were less than 30%, whereas those for patients without these complications were more than 50%. Overall, the functional outcomes were similar between all the reconstructive options, except for those without postoperative major complications, indicating that avoiding these complications is highly important for achieving better functional outcome.

## 4. Discussion

Limb-sparing surgery for periacetabular tumors is one of the most challenging procedures for orthopaedic oncologists [6, 14, 15, 19]. While many surgical options after resection of periacetabular tumors have been reported [3, 27, 28] and several clinical studies have attempted its functional outcomes [14, 20, 21], no standard procedures have been determined. Usually, the technique selected for reconstruction is individualized for each patient. As the choice of the optimal technique for reconstruction after acetabular tumor resection depends on numerous parameters, surgeons need to understand the details of each case and the appropriate indications for each of the reconstructive procedures. In this study, we overviewed the clinical characteristics and postoperative functions in a series of patients who underwent various procedures in a single referral center and found that avoiding postoperative complications is highly important for achieving better function, suggesting that surgical procedures with adequate soft tissue coverage or without the massive use of nonbiological materials are preferable.

Pelvic reconstruction with an endoprosthesis has been a major challenge and in this series the functional results were not satisfactory. Postoperative function was markedly affected by major complications such as deep infection or implant dislocation. To date, various reports have demonstrated the high major complication rates by endoprosthetic replacement, ranging 18–65%; 40–65% in saddle prostheses [29–31], 28–41% in hemipelvic endoprosthesis [20, 21], 25–60% in custom-made hemipelvic endoprosthesis [4, 5, 32], and 18–58% in the other types of endoprosthesis such as ice cream cone endoprosthesis [33], modular hemipelvic endoprosthesis [18], PAR hemipelvic endoprosthesis [34], MUTARS hemipelvic endoprosthesis [35], and constrained hip reconstruction cup (C-THA) endoprosthesis [6]. The high rates of complications, and the associated poor functional results, have been considered to be related to the lack of soft tissue coverage, resection of muscles, and the creation of a large dead space [14]. In our series, patients with major complications also seemed to be attributable to the amount of remaining bone and soft tissues or a dead space. In two recent cases, relatively better function was achieved without postoperative complications using a rectus abdominis myocutaneous (RAM) flap, which is recommended for providing an adequate tissue mass to eliminate the dead space and for covering any exposed bone or implants with well vascularized tissue [26]. Therefore, reconstruction with adequate soft tissue coverage could avoid postoperative complications and improve clinical outcomes of endoprosthetic reconstruction. Recently, an extended application of computer navigation-assisted resection in pelvic tumors has been described [36–38]. Successful case reports have indicated that incorporating computer navigation may aid in accurate intraoperative identification of tumor extent and facilitate bone resections with clear surgical margins in musculoskeletal tumor surgery. Furthermore, CT and MRI fusion images when combined with surgical navigation help surgeons produce a reliable preoperative plan and may improve identification of margins on planned resections, thus avoiding unnecessary resection in musculoskeletal tumor surgery [38, 39]. Therefore, these novel techniques or carefully considered selection criteria, including patients with adequate normal bone and soft tissue after resection or who are suitable for RAM flap, could probably improve the outcome of endoprosthetic reconstruction. Alternately, surgical procedures other than endoprosthetic reconstruction appear to be reasonable options for many of these patients, considering the high complication rate and modest functional scores associated with periacetabular reconstructions as shown in previous reports and our current data.

Patients in this series who underwent hip transposition achieved relatively good function. Hip transposition, reported in 1988 by Winkelmann at the University Hospital of Münster, is characterized by a lower incidence of complications and revision surgery in comparison with other reconstructive procedures [7, 8, 14, 40]. The lower complication rate associated with this procedure is attributed to the smaller dead space resulting from a shift of the hip proximally to the pelvis [40]. Postoperative deep infection in one of the patients in this series might have been caused by the use of a Gore-Tex sheet, which is a nonbiological material. A better functional prognosis would have been expected in this patient if simple reconstruction without nonbiological materials had been possible. In general, patients with pelvic sarcoma have a poor prognosis [41, 42]. Therefore, safer procedures with higher success rates and lower complication rates are desirable. Leg-length discrepancy after surgery is the major problem of this procedure [7, 8]. However, Rödl et al. reported that such leg-length discrepancy could be corrected using distraction osteogenesis [40]. They demonstrated that four patients who underwent limb lengthening after long-term survival achieved good average MSTS scores of 73%. Recent report from Okayama University [43] has also demonstrated good to excellent MSTS scores of 60–93% by using postoperative external fixation instead of pelvic cast which was described as the original method [7]. Collectively, hip transposition is a reasonable reconstruction method with lower complication rates for patients with periacetabular tumors.

Iliofemoral arthrodesis achieved the best functional scores in this series, attributable to a good gait performance with a stiff hip. Previous studies have also mentioned the advantage of a durable, pain-free, but stiff hip and less leg-length discrepancy [10, 28]. We consider that iliofemoral arthrodesis is suitable for patients with strenuous activity requirements. One major problem of iliofemoral arthrodesis is a high rate of nonunion (60%) after primary fusion [44]. However, no patients in this series suffered nonunion, and a recent report has indicated a relatively lower nonunion rate of 14% [10]. One of the suggested reasons for nonunion is that a solid iliofemoral fusion is difficult to achieve, since the surface for bone contact is usually small [10]. Therefore, this reconstruction procedure would be most suitable when the proximal osteotomy is at the lower part of the ilium, as shown in Figure 5. One patient in the present series achieved bone union using a vascularized fibular graft. Appropriate indications and use of a vascularized iliac bone block or fibular graft might improve the union rate.

Although the small number of patients who underwent reconstruction with a frozen bone autograft limits the interpretation of our results, we achieved relatively good functional results. However, the patient developed hip osteoarthritis and has complained of continuous pain. Resurfacing total hip arthroplasty may therefore be necessary in the future. As a previous report has indicated, the advantages of this procedure include a perfect fit, the lack of any need for a bone bank, easy attachment of tendons and ligaments, and a related desirable bone stock [24]. However, this procedure has certain disadvantages, such as a high rate of postoperative infection, degeneration of cartilage over time, and therefore late osteoarthritis. Accordingly, the long-term outcome of reconstruction using a frozen bone autograft remains unknown.

We found that patients with major complications had markedly reduced functional scores. Thus, reduction of the postoperative complication rate is highly important to obtain better function. In general, since the patients with pelvic sarcoma have a poor prognosis, complete resection of the tumor as well as reconstruction without postoperative complications is desirable for them. From this viewpoint, we consider that the surgical procedures without massive use of nonbiological materials, including endoprosthesis, are preferable to achieve better function and fewer complications, although the rarity and variability of these tumors preclude a statistical comparison of outcomes. Alternately, appropriate selection of reconstruction procedures for individual patients, considering the amount of remaining bone and soft tissues and novel techniques such as tissue transfer or computer-assisted surgery, would lead to fewer complications and better function.

## 5. Conclusions

This study summarized the clinical outcomes of major reconstructive procedures after resection of periacetabular tumors. Postoperative major complications, including deep infection or hip dislocation, remarkably worsened functional outcome. Endoprosthetic reconstruction failed without adequate soft tissue coverage. Therefore, avoiding postoperative complications is highly important for achieving better function, suggesting that surgical procedures with adequate soft tissue coverage or without the massive use of nonbiological materials are preferable. Appropriate selection of reconstruction procedures for individual patients, considering the amount of resection and remaining bone and soft tissues, would lead to fewer complications and better function.

## Figures and Tables

**Table 1:** Patient characteristics for the entire study population and surgical/oncological outcome.

Description	Number
Patients	
Male 13, female 5	Total 18
Age (at diagnosis)	41 years (8–69)
Tumor size	11.7 cm (7–20)
Diagnosis	
Osteosarcoma	8
Chondrosarcoma	5
MFH of bone	2
Ewing sarcoma	2
Fibrosarcoma of bone	1
Neoadjuvant therapy	
Polychemotherapy	11
Radiotherapy	0
Adjuvant therapy	
Polychemotherapy	9
Radiotherapy	1
Follow-up	62 months (8–155)
Resected area (Enneking classification)	
PII	2
PI-II	4
PII-III	8
PI-II-III	4
Surgical outcome	
Wide margin	17
Marginal margin	0
Intralesional margin	1
Oncological outcome	
No evidence of disease (NED)	11
Alive with disease (AWD)	2
Dead of disease (DOD)	5
Prognosis	
Overall survival (five years)	67.2%

**Table 2:** Complications according to surgical treatment.

Surgical procedures	Number of patients	Number of patients with major complications	Complications (number)	Local recurrence (number)
Endoprosthesis	11	6 (55%)	Deep infection (2), superficial infection (2), dislocation (2), wound complication (3), abdominal hernia (1)	1
Other reconstructions	7	1 (14%)		0
Hip transposition	4	1 (25%)	Deep infection (1), superficial infection (1), leg-length discrepancy (4)	0
Iliofemoral arthrodesis	2	0	Implant breakage (1), leg-length discrepancy (2)	0
Frozen autograft	1	0	Osteoarthritis (1), wound complication (1)	0

Total	18	8 (44%)		1

**Table 3:** Functional outcomes according to surgical treatment.

Surgical procedure	Score according to musculoskeletal tumor society (MSTS score) system						
	Pain	Function	Acceptance	Support	Distance	Gait	Total
Endoprosthesis	64 (3.2)	42 (2.1)	46 (2.3)	6 (0.3)	44 (2.2)	42 (2.1)	42 (12.5)
Other reconstructions	82 (4.1)	54 (2.7)	57 (2.8)	17 (0.8)	57 (2.8)	60 (3)	55 (16.4)
Hip transposition	90 (4.5)	50 (2.5)	70 (3.5)	0 (0)	35 (1.75)	50 (2.5)	49 (14.7)
Iliofemoral arthrodesis	80 (4)	60 (3)	50 (2.5)	60 (3)	90 (4.5)	70 (3.5)	68 (20.5)
Frozen autograft	60 (3)	60 (3)	20 (1)	0 (0)	80 (4)	80 (4)	50 (15)

Mean scores	73 (3.6)	48 (2.4)	51 (2.5)	10 (0.5)	49 (2.5)	49 (2.5)	47 (14.2)
